# Supplementary material for: Development of the Intestinal Microbiota of Dairy Calves and Changes Associated with Cryptosporidium spp. Infection in Brazil
Source: Microorganisms. 2024 Aug 23;12(9):1744. doi: 10.3390/microorganisms12091744 (PMC11434485; doi:10.3390/microorganisms12091744)
Supplement: Supplementary file 1 [file microorganisms-12-01744-s001.zip › microorganisms-3062666-supplementary.pdf]

Supplementary Table S1. Percentages of relative abundance of the main phyla and genera of fecal samples from dairy calves in different days of life.

**Phyla**	**2nd day**	**3rd day**	**4th day**
Firmicutes	24.69^{a} ±14.50	37.90^{ab} ±27.72	52.57^{a} ±19.70
Proteobacteria	52.33^{a} ±24.88	28.44^{b} ±22.11	20.58^{b} ±17.77
Fusobacteria	18.41 ±23.20	20.68 ±25.29	2.36 ±3.06
Bacteroidetes	4.27^{a} ±8.64	9.32^{ab} ±14.93	19.49^{b} ±16.28
Actinobacteria	0.31^{a} ±0.57	3.67^{b} ±4.01	4.78^{b} ±5.90
Verrucomicrobia	0.00^{a} ±0.00	0.00^{a} ±0.00	0.20^{b} ±0.39
**Genera**	**2nd day**	**3rd day**	**4th day**
Escherichia/Shigella	33.79^{a} ±20.88	22.97^{ab} ±18.59	16.45^{b} ±16.31
Fusobacterium	18.25 ±23.02	20.43 ±25.06	2.34 ±3.03
Enterobacteriaceae	16.64^{a} ±19.87	3.25^{b} ±6.80	1.25^{b} ±1.16
Lactobacillus	2.97 ±5.66	7.20 ±7.03	9.53 ±16.68
Bacteroides	3.77 ±7.16	3.62 ±3.99	9.85 ±11.29
Lachnospiraceae	2.62^{a} ±5.85	4.22^{ab} ±3.76	8.08^{b} ±8.84
Prevotella	0.16^{a} ±0.64	4.72^{ab} ±12.23	8.67^{b} ±9.35
Faecalibacterium	0.43^{a} ±2.07	0.56^{a} ±1.50	11.33^{b} ±15.34
Peptospreptococcus	0.87^{a} ±1.28	6.67^{b} ±10.78	2.94^{ab} ±6.58
Phascolarctobacterium	0.16^{a} ±0.66	2.43^{a} ±4.72	6.49^{b} ±6.53
Clostridium sensu stricto	8.23^{a} ±8.00	0.65^{b} ±1.27	0.18^{b} ±0.45
Megasphaera	0.03 ±0.13	4.71 ±15.49	4.24 ±6.90
Collinsella	0.29^{a} ±0.56	3.63^{b} ±4.00	4.67^{b} ±5.81
Streptococcus	2.14 ±3.06	4.77 ±13.57	1.02 ±2.67
Butyricicoccus	1.92 ±3.55	2.68 ±2.97	2.34 ±2.09
Sutterella	0.92 ±2.07	1.09 ±1.31	1.56 ±2.98
Lactobacillales	0.59 ±0.86	0.96 ±1.52	0.37 ±0.66
Blautia	0.06^{a} ±0.22	0.03^{a} ±0.05	1.55^{b} ±2.47
Alloprevotella	0.01^{a} ±0.03	0.08^{a} ±0.20	0.66^{b} ±0.90

^{a,b,c,d} Superscript letters differ between days.
